# Pleomorphic rhabdomyosarcoma of the left lower extremity with synchronous gastric and small intestinal metastases presenting as intussusception: a case report

**DOI:** 10.3389/fonc.2026.1889815

**Published:** 2026-07-29

**Authors:** Ruyi Yu, Xin Chen

**Affiliations:** 1 Department of Gastrointestinal Surgery, The Second People's Hospital of Neijiang, Neijiang, Sichuan, China; 2 Department of Gastrointestinal Surgery, The Affiliated Hospital of Southwest Medical University, Luzhou, Sichuan, China

**Keywords:** case report, gastric metastasis, gastrointestinal metastasis, intussusception, pleomorphic rhabdomyosarcoma, small intestinal metastasis

## Abstract

**Background:**

Pleomorphic rhabdomyosarcoma (PRMS) is a rare and highly malignant soft tissue sarcoma in adults. Gastrointestinal metastasis is uncommon, and simultaneous involvement of the stomach and small intestine manifesting as intussusception is extremely rare. Early clinical manifestations are insidious and easily misdiagnosed.

**Case presentation:**

A 62-year-old male patient was admitted on February 8, 2025, due to “postprandial abdominal fullness and discomfort for more than 1 month, aggravated with nausea and vomiting for more than 10 days”. He noticed a mass in his left lower leg in January 2023, and underwent mass resection at an outside hospital in December 2023. Postoperative pathology suggested spindle cell rhabdomyosarcoma (RMS). Early tumor recurrence occurred after surgery, and he was admitted to The Affiliated Hospital of Southwest Medical University in January 2024. PRMS was confirmed by pathological consultation, and he received 3 cycles of anthracycline-ifosfamide (AI) regimen chemotherapy. Due to disease progression, left lower extremity amputation was performed in December 2024. The current admission revealed multiple metastases to the stomach and small intestine complicated with intussusception and intestinal obstruction. Emergency partial small bowel resection was performed, and metastatic PRMS was confirmed by postoperative pathology and Immunohistochemistry (IHC). The patient recovered smoothly after surgery.

**Conclusions:**

Adult PRMS is highly malignant with poor chemosensitivity. Synchronous gastric and small intestinal metastases causing intussusception is extremely rare. This case also highlights the critical impact of fragmented care, specifically the lack of adjuvant therapy post-resection and inadequate systemic staging prior to amputation, on disease progression.​ Clinicians should maintain high vigilance for unexplained gastrointestinal symptoms in patients with a history of sarcoma, establish an early diagnosis based on pathology and IHC, and advocate for continuous, standardized, multidisciplinary management​ to improve prognosis.

## Background

Pleomorphic rhabdomyosarcoma (PRMS) is a highly malignant soft tissue sarcoma that mainly occurs in middle-aged and elderly adults, predominantly in the extremities. It is characterized by significant cellular atypia, active mitosis, strong invasiveness, high metastatic risk, and poor chemosensitivity, leading to an overall poor prognosis. Common metastatic sites include the lungs, bones, and lymph nodes. Gastrointestinal metastasis is relatively rare, and simultaneous involvement of the stomach and small intestine as the initial presentation of acute intussusception is even rarer.

Due to the insidious and non-specific early symptoms of such metastases, patients are often misdiagnosed with common gastrointestinal diseases, resulting in delayed diagnosis and treatment. This paper reports a rare case of synchronous gastric and small intestinal metastases causing intussusception shortly after surgery, chemotherapy, and amputation for left lower extremity PRMS. Combined with literature review, we aim to improve clinicians’ awareness of this rare metastatic pattern.

## Case presentation

A 62-year-old male patient was admitted to the Department of Gastrointestinal Surgery, The Affiliated Hospital of Southwest Medical University on February 8, 2025, due to “postprandial abdominal fullness and discomfort for more than 1 month, aggravated with nausea and vomiting for more than 10 days”.

### Diagnosis and treatment of the primary tumor in the left lower extremity

**Figure 1:**
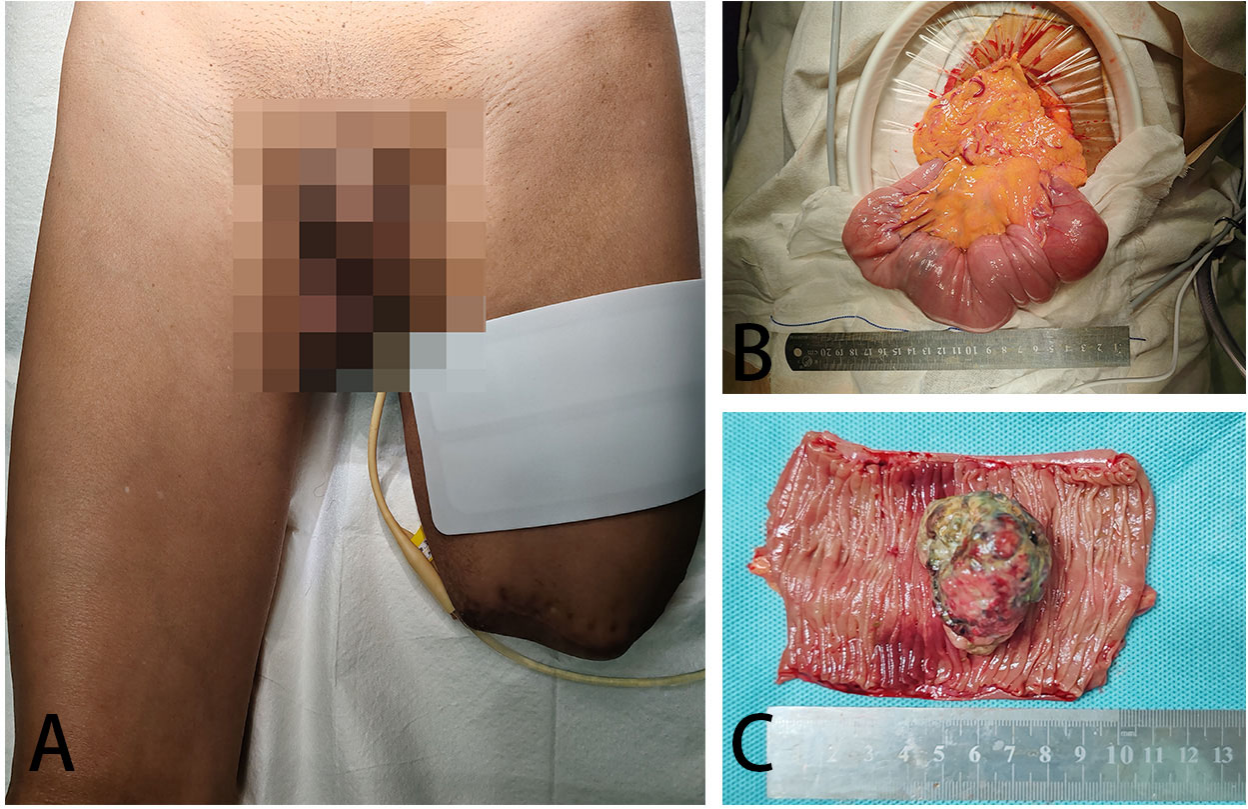
Clinical and intraoperative features of the present case. **(A)** Clinical photograph of the left lower extremity after amputation. **(B)** Intraoperative view showing intussusception of the small intestine. **(C)** Gross appearance of the intraluminal metastatic tumor in the small intestine.

In January 2023, the patient found a mass in his left lower leg without obvious cause, pain, or limited mobility, and received no treatment. In December 2023, he underwent resection of the left lower leg mass at an outside hospital (**Figure 1A**). Postoperative pathology suggested a sarcoma consistent with spindle cell rhabdomyosarcoma (RMS) based on morphology and Immunohistochemistry (IHC), which showed: CD34(-), SMARCA4(+), CK(-), Desmin(+), MDM2(focal+), Ki67(70%+), Myogenin(-), MyoD1(+), NKX2.2(-), SOX10(-), S-100(-), SMA(-), STAT6(-).

**Figure 2:**
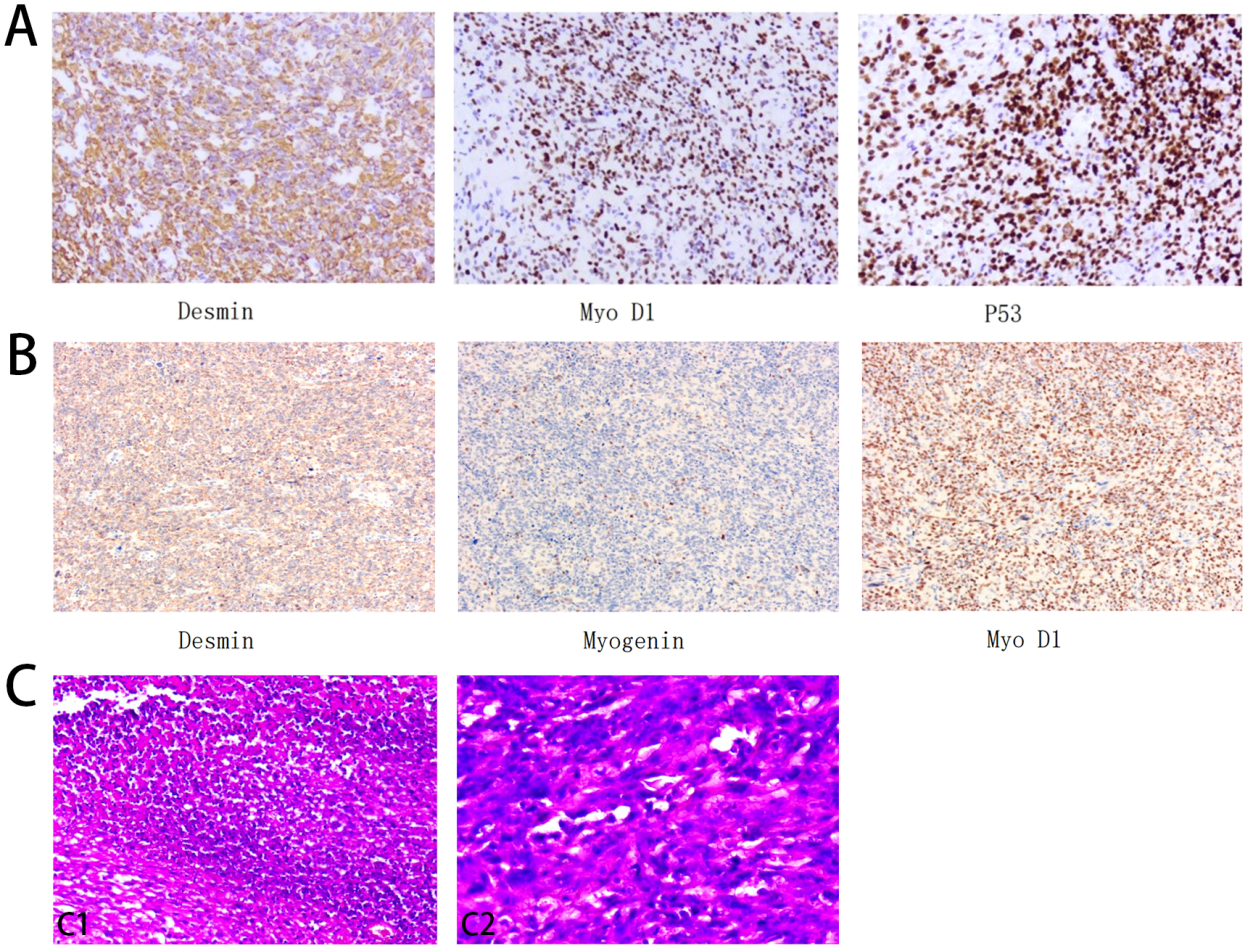
One month after the local excision of the tumor on the left lower limb, the enhanced MRI results of the left lower limb are as follows: **(A)** TW2-TSE signal; **(B)** SETTR-TSE-SENSE signal; **(C)** ET1W-SPIR-SENSE signal; **(D)** TW2-TSE signal.

The patient visited the Department of Oncology, The Affiliated Hospital of Southwest Medical University on January 23, 2024. Physical examination revealed a scar of approximately 6 cm on the anterior shin of the left lower leg with good wound healing. Pathological consultation reported a spindle cell tumor of the left lower leg mass with significant atypia and mitosis in some areas, consistent with PRMS. IHC results: PCK(-), CD34(-), S100(-), SMA(focal+), Desmin(diffuse+), MyoD1(focal+), Myogenin(focal+), P53(strong+, 90%, suggesting mutant type), Ki-67(+, 70%) (**Figure 2A**).

**Figure 3:**
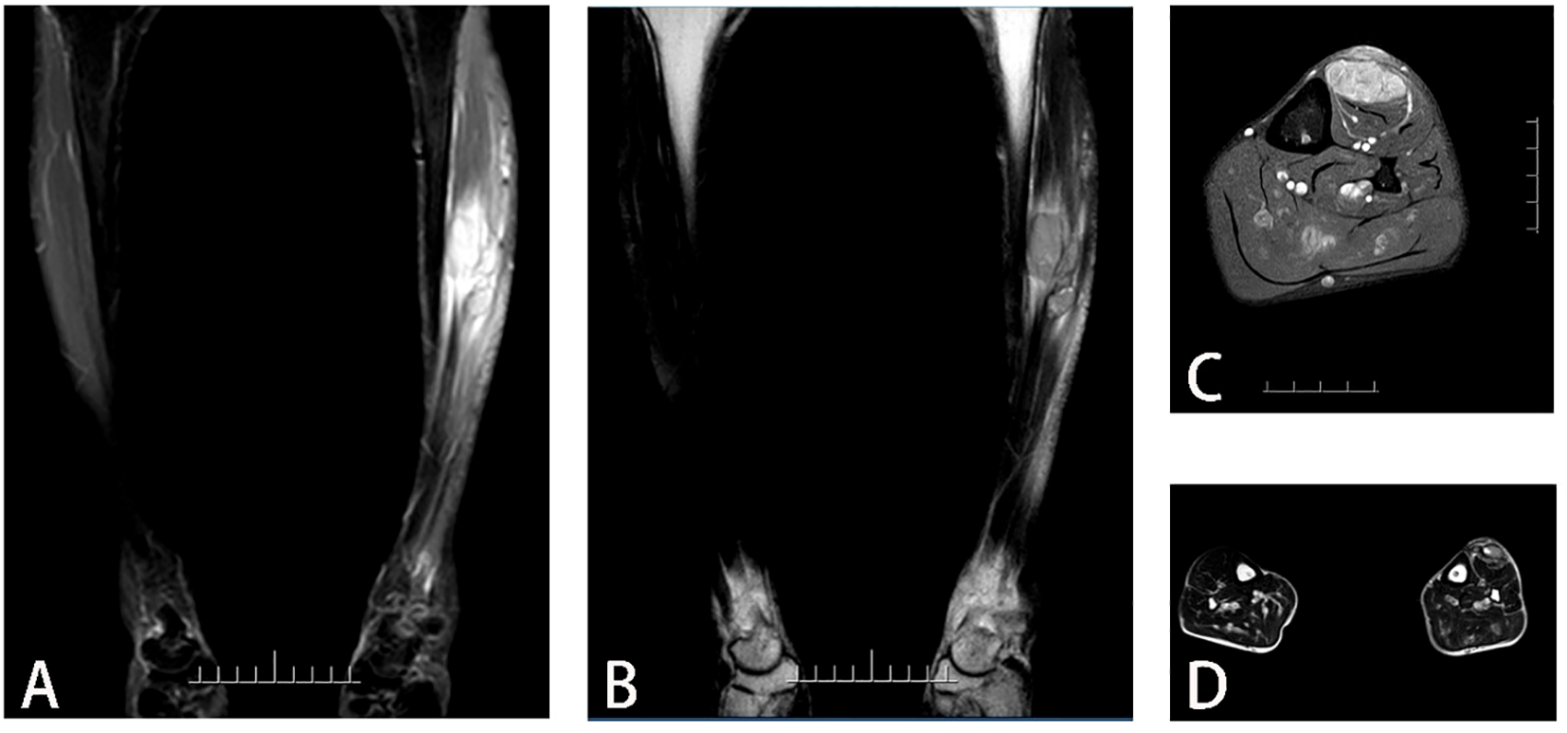
Results of gastroscopy, endoscopic ultrasound, and abdominal contrast-enhanced CT two months after left thigh amputation. **(A, B)** Gastroscopic views revealing metastatic rhabdomyosarcoma in the stomach, showing ulcerative and nodular mucosal lesions. **(C, D)** Endoscopic ultrasound images demonstrating hypoechoic masses consistent with gastric metastasis from rhabdomyosarcoma. **(E, F)** Axial contrast-enhanced CT scans of the abdomen/pelvis showing small bowel obstruction due to metastatic rhabdomyosarcoma, with dilated loops and wall thickening.

Contrast-enhanced MRI of the left lower leg on January 24, 2024, indicated sarcoma recurrence in the extensor hallucis longus muscle of the left lower leg (**Figure 3**). The patient received 3 cycles of anthracycline-ifosfamide (AI) regimen chemotherapy from January to March 2024. Medical records regarding his care between March and December 2024 are unavailable, and it remains unknown whether he received regular medical follow-up or specific therapy during this interval.​ Left lower extremity amputation was ultimately performed in December 2024.

### Diagnosis and treatment during current admission

Physical examination on admission: The abdomen was flat without peristalsis or visible peristaltic waves. Deep tenderness was present in the middle and upper abdomen, without rebound tenderness or muscle rigidity. No obvious mass was palpable. Bowel sounds were 4 times per minute without high-pitched sounds or splashing noises. The left lower extremity was amputated with a well-healed stump.

**Figure 4:**
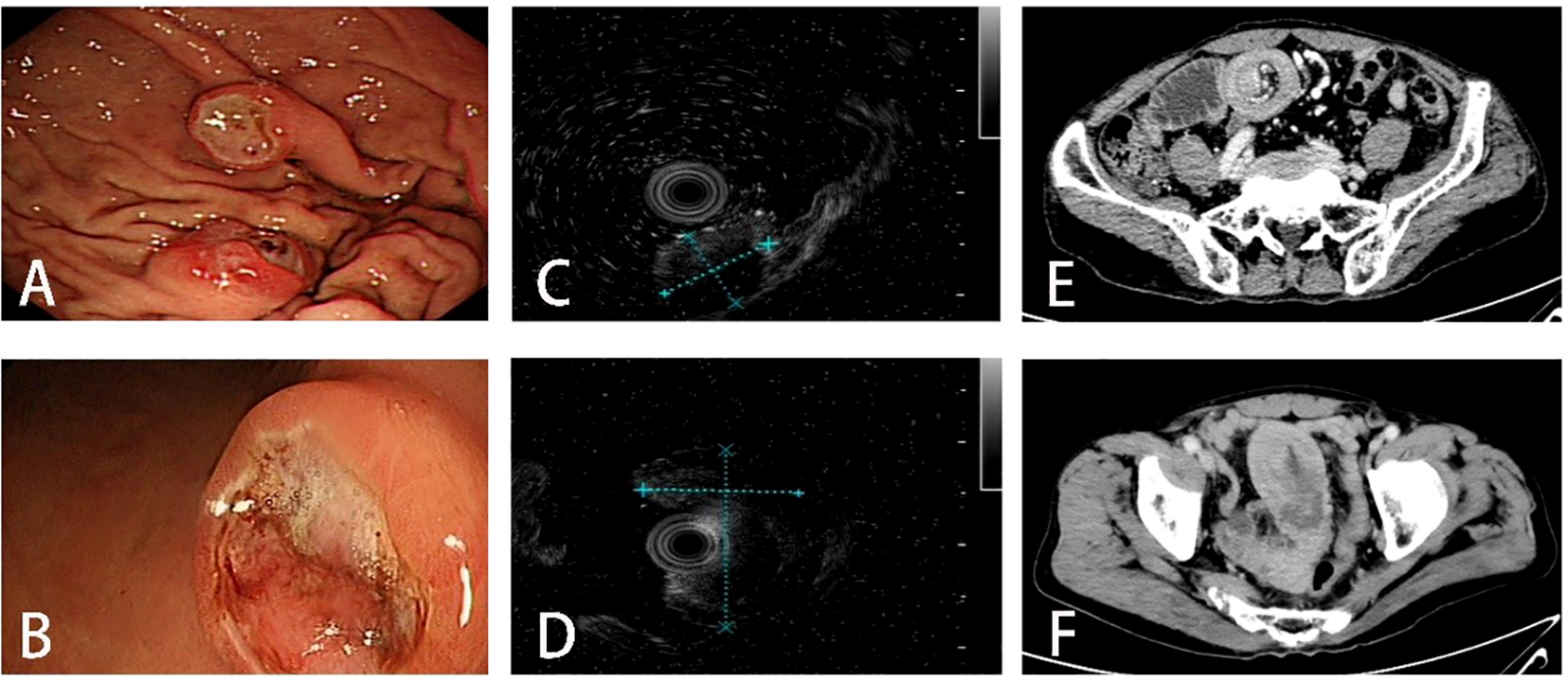
**(A)** Immunohistochemical (IHC): staining of the primary pleomorphic rhabdomyosarcoma (PRMS) after amputation (200× magnification). **(B)** IHC staining of the metastatic pleomorphic rhabdomyosarcoma in the small intestine (100× magnification). **(C)** Hematoxylin and eosin (H&E) staining of the metastatic pleomorphic rhabdomyosarcoma (PRMS) in the small intestine (C1 200× magnification; C2 400× magnification).

Gastroscopy (February 4, 2025): A protruding mass of approximately 2.0 cm×2.5 cm was found in the middle part of the lesser curvature of the gastric body with ulceration; multiple polypoid protuberances with a diameter of 0.6–1.0 cm were seen along the greater curvature of the gastric body (**Figures 4A, B**).

Gastric biopsy pathology: Malignant tumor consistent with metastatic RMS based on IHC.

Endoscopic ultrasonography (February 11, 2025): Multiple protruding lesions with depression, necrosis, and erosion were visualized in the gastric body and the fundus near the greater curvature of the gastric body. The largest lesion in the middle part of the lesser curvature of the gastric body measured about 2×2.5 cm with an ulcerative lesion on the surface. Ultrasonography showed mixed echo masses in the lesions, spindle-shaped, with sectional sizes of 0.7×0.9 cm and 0.9×1.0 cm near the gastric body at the fundus, and a maximum sectional size of 1.1×1.7 cm at the lesser curvature of the gastric body. Mucosa was partially absent at the depressed lesions, with blurred layers of the mucosa, muscularis mucosae, and submucosa. The muscularis propria was intact, with unclear boundaries and heterogeneous internal echoes (**Figures 4C, D**).

Chest and abdominal CT (February 11, 2025): Intussusception in the right lower abdomen and pelvic cavity with proximal small intestinal dilatation, pneumatosis, fluid effusion, and air-fluid levels, indicating intestinal obstruction. The stomach was poorly distended, and no other distant metastases were found (**Figure 4E, F**).

Laboratory examinations: Elevated white blood cells and neutrophils, mild anemia, hypoalbuminemia, elevated CA199, positive fecal occult blood test, and elevated hepatitis B viral load. Coagulation function, renal function, and electrolytes were unremarkable.

The patient had acute intestinal obstruction with emergency surgical indications. Emergency exploratory laparotomy and partial small bowel resection were performed after excluding contraindications.

Postoperative pathological examination: Gross examination: A protuberant mass measuring 4.5×4.0×2.4 cm with grayish-white, solid, and hard cut surfaces, invading the muscular layer, with an unclear boundary to surrounding tissues, growing circumferentially and occupying about two-thirds of the intestinal lumen (**Figure 1B**). The remaining intestinal mucosa was smooth, and no enlarged lymph nodes were detected in the pericolic fat. Microscopy: Tumor lesions with necrosis, marked atypia in some areas, and visible mitosis. Consistent with metastatic RMS (**Figure 1C**). Histopathological examination via H&E staining (**Figure 2C**) revealed pleomorphic rhabdomyosarcoma characterized by prominent nucleoli, frequent mitotic figures, and areas of necrosis. IHC: SMA(-), Desmin(+), MyoD1(+), Myogenin(focal+), P53(+, suggesting missense mutation), Ki-67(+, 80%), CD117(-), CD34(-), S100(-), DOG1(-) (**Figure 2B**).

The patient recovered smoothly after surgery with stable vital signs and gradual resumption of oral intake. No complications such as abdominal pain, distension, or fever occurred. The incision healed well. He was discharged 10 days after surgery with normal review indicators. Prior to discharge, the patient was discussed at a multidisciplinary tumor board (MDT) comprising gastrointestinal surgery, medical oncology, radiology, gastroenterology, and pathology. The MDT concluded that the acute small bowel intussusception with obstruction required emergent surgical intervention, whereas the synchronous gastric metastases, being multifocal submucosal lesions, were not amenable to curative−intent resection and were best managed with palliative systemic therapy and endoscopic surveillance. Although no standardized regimen currently exists for advanced PRMS, recent data from the global Pushing Ultra-Rare Sarcomas Beyond Hope (PUSH) consortium support the use of gemcitabine−based combinations or anthracycline−based chemotherapy, with pazopanib representing a potential option in appropriately selected patients. Close follow−up with repeat gastroscopy and contrast−enhanced CT was recommended to monitor treatment response and detect early progression, and the patient was scheduled for further multidisciplinary comprehensive treatment and regular follow−up.

## Discussion and conclusions

Pleomorphic rhabdomyosarcoma (PRMS) is a highly malignant soft tissue sarcoma originating from cells with skeletal muscle differentiation, characterized by marked cellular pleomorphism (1). It predominantly affects middle-aged and elderly individuals aged 40–70 years, with a peak incidence at approximately 60 years, and most commonly involves the extremities (1). As an extremely rare subtype, PRMS accounts for a very small proportion of adult soft tissue sarcomas and exhibits highly aggressive biological behavior. According to the 2020 World Health Organization (WHO) classification of soft tissue and bone tumors, rhabdomyosarcoma (RMS) is mainly classified into embryonal, alveolar, pleomorphic, and spindle cell/sclerosing subtypes. Among these, PRMS and spindle cell/sclerosing RMS occur mainly in adults and are associated with significantly poorer prognosis than embryonal and alveolar subtypes, which are more common in the pediatric population. Pathologically, PRMS is featured by prominent tumor cell atypia, active mitosis, and frequent necrosis. Immunohistochemically, it is characterized by the expression of myogenic markers including Desmin, MyoD1, and Myogenin, which enable differential diagnosis from other undifferentiated or pleomorphic sarcomas. Meanwhile, P53 mutation and high Ki-67 expression indicate high proliferative activity, strong invasiveness, and poor chemotherapeutic response, which are consistent with the immunophenotypes of both the primary and metastatic lesions in the present patient.

PRMS is highly malignant with a propensity for early local invasion and distant metastasis. Hematogenous metastasis is the predominant route, with common metastatic sites being the lungs, bones, and regional lymph nodes in sequence. Gastrointestinal metastasis is relatively rare, and synchronous involvement of the stomach and small intestine presenting as acute intussusception and intestinal obstruction is extremely rare. The rich blood supply and close connection between the draining veins and systemic circulation in the gastrointestinal tract provide an anatomical basis for hematogenous seeding of highly malignant sarcomas. However, due to insidious early onset and non-specific symptoms, metastatic lesions mostly present as non-specific symptoms such as upper abdominal discomfort, dull pain, abdominal distension, and melena, which can be easily misdiagnosed as gastritis, peptic ulcer, gastrointestinal stromal tumor, or primary gastric cancer. The patient had a 2-year history of primary PRMS in the left lower extremity, and the disease progressed continuously despite surgical resection, chemotherapy, and amputation. He was admitted recently due to new-onset upper abdominal fullness, nausea, and vomiting. Gastroscopy and endoscopic ultrasonography revealed multiple gastric protruding lesions with necrosis and ulceration, which were difficult to differentiate from primary gastrointestinal malignancies preoperatively. The final diagnosis of gastrointestinal metastasis was confirmed by combining the previous history of soft tissue sarcoma, pathological morphology, and characteristic myogenic immunohistochemical markers, suggesting that clinicians should promptly screen for distant metastasis in sarcoma patients with new-onset gastrointestinal symptoms.

The treatment of RMS is based on multidisciplinary comprehensive therapy combining surgery, chemotherapy, and radiotherapy (1–5). For localized PRMS, the surgical goal is complete resection with negative margins (1). For cases with repeated local recurrence or non-salvageable extremities, amputation remains an important approach to control local lesions. Nevertheless, PRMS possesses inherent chemoresistance, and its sensitivity to conventional chemotherapy is significantly lower than that of embryonal and alveolar subtypes. Early recurrence and distant metastasis frequently occur even after standard perioperative chemotherapy. Notably, a recent global, multicenter, retrospective study from the Pushing Ultra-Rare Sarcomas Towards Hope (PUSH) consortium (6), which included 77 adult patients with advanced PRMS and represents the largest cohort to date, demonstrated that anthracycline-based regimens achieved an objective response rate (ORR) of 50%, with median progression-free survival (mPFS-2) and median overall survival (mOS-2) of 5.2 and 19.2 months, respectively. Gemcitabine-based regimens also showed meaningful efficacy with an ORR of 42%, and this study represents the first report demonstrating the antitumor activity of gemcitabine alone or in combination with docetaxel in advanced PRMS. Pazopanib and PD-1 inhibitors yielded anecdotal responses. Consistent with these findings, the present patient exhibited disease progression despite 3 cycles of anthracycline-ifosfamide (AI) chemotherapy, further supporting the inherent chemoresistance of PRMS. Radiotherapy is generally not recommended for gastrointestinal metastatic lesions due to the high risk of complications. In recent years, targeted therapy and immunotherapy have been gradually explored, but no standard therapeutic regimen has been established.

The prognosis of adult PRMS is generally poor. Major adverse prognostic factors include advanced age, large tumor size, high histological grade, distant metastasis at diagnosis, chemoresistance, and positive surgical margins. Compared with pediatric RMS, whose 5-year survival rate exceeds 80%, the 5-year survival rate of adult patients is only 27%–40%, and the prognosis is even worse for those with metastatic disease (2). The patient developed extensive gastrointestinal metastasis within only 2 years after initial diagnosis, and the disease progressed rapidly despite multiple interventions including surgery, chemotherapy, and amputation, further confirming the highly aggressive and dismal prognostic characteristics of PRMS. Meanwhile, synchronous gastric and small intestinal metastasis manifesting as intussusception is extremely rare and difficult to recognize clinically, suggesting that clinicians should strengthen the awareness of rare metastatic sites, incorporate gastrointestinal evaluation into the routine follow-up monitoring of patients with advanced soft tissue sarcoma, and establish a definite diagnosis as early as possible via endoscopy, imaging, and pathological biopsy.

Multidisciplinary team (MDT) discussion played a pivotal role in the management of this case. The MDT, comprising gastrointestinal surgery, medical oncology, radiology, gastroenterology, and pathology, concluded that the acute small bowel intussusception with obstruction required emergent surgical intervention. In contrast, the synchronous gastric metastases were not amenable to curative-intent resection, as they presented as multifocal submucosal lesions. These lesions were best managed with palliative systemic therapy and endoscopic surveillance. Although no standardized regimen currently exists for advanced PRMS, recent data from the global PUSH consortium support the use of gemcitabine-based combinations or anthracycline-based chemotherapy, with pazopanib representing a potential option in appropriately selected patients. Close follow-up with repeat gastroscopy and contrast-enhanced CT was recommended to monitor treatment response and detect early progression.

Beyond the inherent tumor biology, this case also highlights the critical importance of continuous and standardized multidisciplinary management​ in high-grade sarcomas. The patient received fragmented care across multiple institutions, with a significant gap in documented follow-up and systemic therapy between March and December 2024. This discontinuity poses a major challenge to disease monitoring and timely intervention. While the aggressive nature of PRMS is the primary driver of the poor outcome, the lack of standardized care during critical intervals may have contributed to the delay in detecting progression, ultimately leading to widespread metastatic disease. Therefore, ensuring seamless transitions of care and strict adherence to follow-up protocols is paramount to improving the prognosis of patients with highly malignant soft tissue sarcomas.

In summary, PRMS is an extremely rare, highly malignant, and chemoresistant soft tissue sarcoma in adults with a high tendency for early recurrence and distant metastasis. Synchronous gastric and small intestinal metastasis presenting as intussusception is particularly rare, characterized by insidious early symptoms and a high misdiagnosis rate. While this case is marked by limitations, specifically the absence of adjuvant therapy post-resection and inadequate systemic staging prior to amputation,​ these shortcomings underscore the critical importance of standardized management. Clinicians should deepen the understanding of the biological behavior and rare metastatic patterns of this disease. Furthermore, this case reinforces the necessity of establishing continuous, standardized, and multidisciplinary management systems for sarcoma patients to avoid fragmented care and ensure timely intervention. For patients with a history of soft tissue sarcoma, once new-onset unexplained gastrointestinal symptoms appear, endoscopy, imaging, and pathological examination should be performed as soon as possible to avoid missed diagnosis and misdiagnosis. Multidisciplinary collaboration should be applied in treatment, with surgery as the mainstay, combined with chemotherapy and radiotherapy rationally. Meanwhile, novel therapeutic strategies should be actively explored to improve the prognosis of such rare and high-risk patients.

## Declaration of case limitations

This study has several limitations primarily stemming from fragmented care across multiple institutions. First, the absence of adjuvant radiotherapy or chemotherapy following the initial wide local excision in December 2023 represents a deviation from standard sarcoma management protocols. Second, a comprehensive systemic evaluation (e.g., whole-body staging with PET-CT or thoracoabdominal CT) was not performed prior to the amputation surgery in December 2024, potentially allowing micro-metastases to progress undetected. Additionally, the unavailability of histopathology slides from the 2024 amputation specimen precluded a full comparative analysis across all time points. These gaps in documentation and clinical management limit our ability to precisely delineate the trajectory of disease progression.

## Data Availability

The clinical data generated in this case report contains protected health information (PHI) and is subject to patient privacy regulations and institutional data protection policies. No publicly accessible repository is used, and the data is not included in the article or supplementary materials. The authors are unable to share individual patient-level data due to ethical and legal restrictions. Requests to access the datasets should be directed to gi_surgeon9527@163.com.
